# Assessment of Pharmaceuticals, Personal Care Products, and Hormones in Wastewater Treatment Plants Receiving Inflows from Health Facilities in North West Province, South Africa

**DOI:** 10.1155/2018/3751930

**Published:** 2018-10-30

**Authors:** Kwangu M. Kanama, Adegbenro P. Daso, Lizzy Mpenyana-Monyatsi, Marthie A. A. Coetzee

**Affiliations:** Department of Environmental, Water and Earth Sciences, Faculty of Science, Tshwane University of Technology, Private Bag X680, Pretoria 0001, South Africa

## Abstract

The presence of 17 pharmaceutical and personal care products (PPCPs) belonging to various therapeutic categories was investigated in two hospital wastewater treatment plants (WWTPs) in North West Province, South Africa. The compounds were extracted from wastewater samples by solid-phase extraction and analysed by liquid chromatography-tandem mass spectrometry. The results showed that ofloxacin, chloramphenicol, and bezafibrate were generally below the limit of quantification (LOQ) in the analysed samples. Acetaminophen and ibuprofen were the dominant pharmaceuticals in the influent streams with corresponding concentrations ranging from 21 to 119 *μ*g/L and 0.3 to 63 *μ*g/L, respectively. Both WWTPs were shown to have the capability to remove some of the target PPCPs, including acetaminophen (76-98%), tetracycline (15-93%), ibuprofen (44-99%), and triclocarban (13-98%). The monitoring of the target PPCPs in both influent and effluent samples of the investigated WWTPs revealed that the discharge of inadequately treated effluents could be contributing to the possible increase in the concentrations of these contaminants in the receiving environmental compartments. Further studies must be focused on the broader characterisation of these matrices in order to assess the potential ecological impacts of this waste disposal practice.

## 1. Introduction

The widespread use of pharmaceuticals and personal care products and their distribution and occurrence in sewage effluents have been extensively reported [1–4]. A number of therapeutic pharmaceuticals are used in large quantities and may be present in influents and treated effluents at varying concentrations ranging from micrograms per litre to nanograms per litre [5–7]. Wastewater emanating from health care facilities is known to contain nonmetabolised pharmaceutical compounds, including antibiotics, anaesthetics, disinfectants, radioactive elements and X-ray contrast agents, among others [8–10]. In addition, these bioactive compounds, both in their unchanged forms or as metabolites or conjugates, are excreted from the human body in urine and faeces, and often end up in the sewer system, which remains the major route of entry into the municipal sewage systems [11–13]. In most cases, these contaminants are not adequately removed during wastewater treatment processes [5, 14, 15]. Thus, their presence in various environmental media signifies a potential danger to public health, biota, and the environment [16, 17]. As a result of their unique physicochemical properties (polarity, water solubility, microbial resistance, and persistence) [18, 19], these bioactive chemicals also exhibit the potential to bioaccumulate in the food chain [20].

The ubiquitous occurrence of pharmaceuticals in hospital wastewater (HWW) has been confirmed in several studies all over the world (Table 1). The concentrations of pharmaceuticals varied among the different hospital wastewater and concentrations varied from nanogram per litre to microgram per litre. The average concentrations for the different classes of compounds are recorded in Table 1.

Wastewater treatment plants (WWTPs) play an important role in minimising the levels of harmful contaminants in reclaimed water, although their complete elimination by the conventional wastewater treatment processes remains unrealistic [39], particularly in the case of the polar organic pollutants because of their relatively high aqueous solubility [36, 40]. The removal rate of some pharmaceutical compounds during wastewater treatment processes is quite low and therefore they have been detected in surface water, groundwater, and drinking water samples [8, 41, 42]. Studies conducted on the Umgeni River in KwaZulu-Natal, South Africa, reported the occurrence of antibiotic, antipyretic, antiepileptic, antipsychotic drug residues, and caffeine in the analysed surface water and sediment. However, most of these pharmaceutical residues were generally detected at a concentration lower than 10 *μ*g/L in surface water, except for the antipyretics, which were generally detected at higher concentrations [43, 44].

To the best of our knowledge, no studies have been reported on the concentrations of pharmaceuticals in wastewater emanating from hospitals in North West Province, and in hospital effluents of the entire country as a whole. Therefore, the aim of this study was to determine the concentrations of a wide range of pharmaceuticals (nonsteroidal anti-inflammatory drugs, beta-blockers, antibiotics, lipid regulating agents, disinfectants, and hormones) in influents and effluents at two hospital WWTPs in North West Province and to evaluate the efficiency of the investigated WWTPs to remove these emerging organic contaminants (EOCs). The selected target compounds represent a broad range of chemicals with different physicochemical properties as shown in Table 2.

## 2. Materials and Methods

### 2.1. Reagents and Chemicals

Pure standards (>98%) of ketoprofen (KET), ibuprofen (IBU), bezafibrate (BF), triclosan (TCS), triclocarban (TCC), chloramphenicol (CAL), norfloxacin (NOR), ofloxacin (OFL), ciprofloxacin (CIP), acetaminophen (ACE), atenolol (ATE), tetracycline (TCN), diclofenac salt (DIC), estrone (E1), 17*β*-estradiol (E2), estriol (E3), and 17*α*-ethinylestradiol (EE2) were all purchased from Sigma-Aldrich (Aston Manor, South Africa). In addition, ^13^C_3_-caffeine (employed as the isotopically labelled surrogate standard), norfloxacin-d5 (internal standard), and 17*β*-estradiol ^13^C_3_ (internal standard) were also obtained from Sigma-Aldrich (Aston Manor, South Africa). Acetone (HPLC grade), LC-MS grade water and methanol (99% purity), formic acid (99% purity), ammonium acetate, and ammonium hydroxide were also all obtained from Sigma-Aldrich (Aston Manor, South Africa). Whatman glass fibre filter paper (pore size 0.7 *μ*m) and SPE cartridges Oasis HLB (500 mg, 12 mL) were purchased from Sigma-Aldrich (Aston Manor, South Africa).

### 2.2. Sample Collection and Preparation

**Figure 1:**
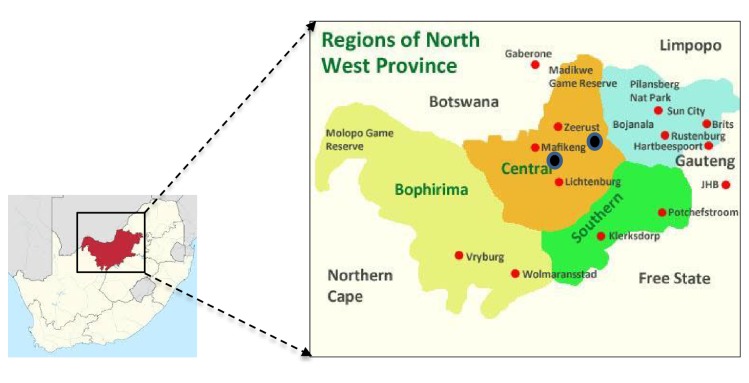
Map showing the North West Province and the sampling points depicted by the black dot.

The wastewater samples were collected from two WWTPs receiving wastewater from hospitals in Ngaka Modiri Molema District, North West Province, South Africa. Both WWTP-A and WWTP-B mainly receive wastewater from a hospital and a clinic, respectively. Below is the map depicting the two sampling locations in relation to the rest of the North West Province and its neighbouring provinces (Figure 1).

In both WWTPs, the treatment process consists of grit channels, aeration tanks, a secondary sedimentation tank, maturation pond, and sludge dewatering units. The average daily flow in WWTP-A and WWTP-B is 322 m^3^/day and 238 m^3^/day, respectively. During August 2015 and December 2015, grab samples were collected from influent and effluent streams at both WWTPs using precleaned 2.5 L amber glass bottles. Samples were collected in duplicate at each site. About 10 mL of formaldehyde (1%, v/v) was immediately added to the amber glass bottles on site to prevent degradation and these samples were kept in the ice box during transportation to the laboratory. In the laboratory, the bottles were kept in the dark at 4°C for less than 72 h until extraction.

*Preparation of Standard Solutions*. Stock solutions of individual pharmaceuticals (5 000 mg/L) were prepared from which multistandards of different concentrations were prepared daily by appropriate dilution of the stock solutions using methanol. Bezafibrate, ibuprofen, acetaminophen, triclosan, atenolol, ketoprofen, tetracycline, 17*β*-estradiol, 17*α*-ethinylestradiol, and 17*β*-estradiol ^13^C_3_ were prepared in methanol. Triclocarban was prepared in a mixture of methanol/acetone (1:1, v/v); ofloxacin was prepared in a mixture of methanol/acetic acid/methanol: water (1:2.5:9 v/v). Ciprofloxacin was prepared in a mixture of 0.1 N hydrochloric acid/methanol: water (4:1 v/v). The stock solutions were stored in amber glass bottles at 4°C. Estrone and estriol were prepared in a mixture of methanol/dichloromethane (1:4, v/v).

### 2.3. Solid-Phase Extraction

The extraction methods of pharmaceutical compounds (antibiotics, disinfectants, beta-blockers, and analgesics) were modifications of the methods presented by Langford et al. [34] and Dorival-Garcia et al. [47]. Briefly, influent and effluent samples were filtered through GF/F filters (0.7 *μ*m). Filtered wastewater samples were then spiked with the surrogate standard (1 mL of 200 *μ*g/L of ^13^C_3_-caffeine). Surrogate standards were added to each sample to account for any potential loss during the sample extraction. Spiked samples were allowed to equilibrate for 60 min. The SPE cartridges (Oasis HLB, 12 mL, 600 mg) were conditioned with 6 mL of methanol and 6 mL of Milli-Q water before the samples (250 mL) were transferred to the cartridges. Thereafter, the sample cartridges were rinsed with 6 mL of distilled water and dried for 20 min under vacuum. The analytes on the dried cartridge were eluted with 6 mL of methanol, and the extracts were evaporated to dryness under a gentle nitrogen stream. The dried extract was reconstituted with 1 000 *μ*L of methanol (for target compounds monitored in negative ESI mode) and 1 000 *μ*L norfloxacin-d5 (100 *μ*g/L) (for target compounds monitored in positive ESI mode), respectively. In all cases, each sample was analysed in triplicate.

The extraction method of steroid hormones followed a method previously presented by Li et al. [48], with some modifications. Briefly, influent and effluent samples were filtered through GF/F filters (0.7 *μ*m). The SPE cartridges (Oasis HLB, 12 mL, 600 mg) were conditioned with 6 mL of methanol and 6 mL of Milli-Q water, and 250 mL of the sample was slowly loaded onto the cartridges. The sample containers were rinsed with 6 mL of Milli-Q water and dried for 20 min under vacuum. The analytes on the dried cartridges were eluted with 12 mL of methanol, and the extracts were evaporated to dryness under a gentle nitrogen stream. The dried extract was reconstituted with 1 000 *μ*L of ^13^C_3_-17*β*-estradiol (500 mg/L). In all cases, each sample was analysed in triplicate.

### 2.4. LC-MS/MS Analysis

In this study, all the target compounds were classified into three groups and various analytical techniques were used to identify the compounds. Group 1 was composed of pharmaceuticals, namely ciprofloxacin, norfloxacin, tetracycline, diclofenac sodium salt, ofloxacin, acetaminophen, and atenolol, which were analysed in positive ESI mode. Group 2 consisted of pharmaceuticals and personal care product compounds, including ketoprofen, ibuprofen, bezafibrate, triclosan, and chloramphenicol, and were analysed in negative ESI mode, while Group 3 was composed of the steroid hormones, namely, estrone, estriol, 17*β*-estradiol, and 17*α*-ethinylestradiol (EE2), which were analysed separately in negative ESI mode. The target compounds in Groups 1 and 2 were chromatographically separated on a Supelco Titan™ C18 UHPLC column (1.9 *μ*m particle size, 5 cm x 2.1 mm), which was locally supplied by Sigma-Aldrich (Aston Manor, South Africa). In all cases, an injection volume of 10 *μ*L was used and the column was maintained at 40°C. A constant flow rate of 0.3 mL/min and the mobile phases consisted of 20 mM ammonium acetate in water (A) and 100% methanol (B) for the analysis of Group 1 candidates. The gradient elution program was as follows: 90% (A) for 1 min, then from 90% to 30% (A) for 3 min, which was further lowered to 10% (A) for 6 min; 9 min, 0%; 12 min, 10%; 15 min, 30%; 15.01 min, 90%; 30 min, 90%. For the Group 2 candidates, the mobile phases consisted of 0.1% formic acid in water (A) and 100% methanol (B). The column was maintained at 40°C and the flow rate was maintained at 0.2 mL/min. The gradient elution program was as follows: 90% (A) for 1 min, then from 90% to 20% (A) for 3 min, which was further lowered to 5% (A) for 6 min; 9 min, 0%; 10 min, 5%; 12 min, 20%; 15 min, 90%; 20 min, 90%.

The target steroid hormones investigated in this study were chromatographically separated on an InertSustain C18 column (3 *μ*m particle size, 2.1 x 150 mm) (Tokyo, Japan). In this case, an injection volume of 10 *μ*L was employed throughout the analysis. The column was maintained at 40°C at a flow rate of 0.3 mL/min. The mobile phases employed consisted of 0.1% NH_4_OH in water (A) and 0.1% NH_4_OH in methanol (B). The gradient elution program was as follows: 50% (A) for 0.01 min; then from 50% to 20% (A) for 3 min; 12 min, 20%; 15 min, 20%; 17 min, 50%; and 20 min, 50%.

Mass spectrometry measurement was performed using an LCMS-8030 model (Shimadzu, USA), which was equipped with an electrospray ionisation source. The source heating block was maintained at 400°C, while the desolvation temperature of 250°C was employed. Nitrogen was used as the drying and nebulising gas (1.50 L/min), while the collision-induced dissociation (CID) gas was argon and was maintained at 230 kPa. The resulting fragment ions were monitored in multiple reaction monitoring (MRM) mode with a dwell time of 100 milliseconds. The details of the precursor and product ions and their collision energies as well as cone voltages are presented in Table 3. The MRM transitions for the individual target compounds were obtained by direct infusion of 1 mg/L of each compound at a flow rate of 0.3 mL/min into the mass spectrometer. Upon the completion of these preliminary experiments, the precursor and product ions of each compound were identified. The details of the MRM transitions for each compound as well as the parameters that were optimised are presented in Table 3.

*Quality Assurance and Quality Control*. Due to the nonavailability of appropriate surrogate standards for the target compounds, spiking experiments were performed to assess the accuracy of the analytical protocol employed. In this case, triplicate analysis of spiked Milli-Q water (250 mL) at two concentrations corresponding to low (10 *μ*g/L) and high (100 *μ*g/L) levels were employed. The estimated recoveries of the target compounds (hormones) ranged from 60 to 150%, whereas low recoveries were observed for pharmaceuticals (below 40%). The low recovery of pharmaceuticals is due to the use of small volume of eluent on high capacity of cartridges (12 mL) similar to that recommended in the USEPA's method 1694 for the analysis of pharmaceuticals and personal care products in water, soil, sediment, and biosolids by HPLC-MS/MS. Incidentally, the adsorbent mass in the cartridges employed for the present study was more than those employed in the USEPA's method. The analytical recovery of the target compounds as well as other method validation parameters is summarised and presented in Tables 4 and 5. During the analysis, reagent and procedural blanks were simultaneously analysed with the extracted samples to assess possible sources of contamination. The linearity of the calibration plots exceeded 0.99 for all the target compounds. The limit of detection (LOD) and the limit of quantification (LOQ), which were derived from the calibration plots, were defined as 3.3 and 10 times the standard deviation (SD) of the blank, respectively, and the residual standard deviations of the resulting calibration curves ranged from 1 to 101.92%. The chromatograms of the targeted compounds are attached in the list of annexure.

*Statistical Analyses*. Statistical analysis was performed by means of the Mann-Whitney U test in order to determine the significance of the differences found between the average concentrations in the effluent and the inlet streams of the two WWTPs.

## 3. Results

The concentrations of the seventeen (17) target pharmaceuticals in the influent streams of both WWTPs are presented in Table 6 and Table 7. Overall, 14 of the 17 target PPCPs were detected. In the influent samples, significant variations in the concentrations of the target PPCPs were observed at the two investigated WWTPs. The observed variations could be due to the temporal variations in the utilisation of prescribed drugs at both hospitals. For instance, analgesic/anti-inflammatory drugs (ACE and IBU) and the antibiotic (TCN) were the most abundant compounds in the influent streams of both WWTP-A and WWTP-B. In contrast, OFL, CAL, and BF were not detected in the influents of the investigated WWTPs.

The presence of emerging organic contaminants, particularly pharmaceuticals and steroid hormones in WWTP effluents, and their subsequent removal during wastewater treatment processes are influenced by several factors. Among these, their aqueous solubility, volatility, adsorption to solids, among others as well as their tendency to undergo biodegradation in aqueous waste streams are important factors that may significantly influence their environmental fate and behaviour [49]. In the present study, the removal efficiencies of both WWTPs for the targeted pharmaceuticals and hormones were calculated employing

(1) $$\begin{array}{c} Removal\;\;efficiency\;\;\%=\frac{Cinfluent-Ceffluent}{Cinfluent}x100 \end{array}$$

where* C*_influent_ and* C*_effluent_ represent the mean concentrations in influent and effluent, respectively

**Figure 2:**
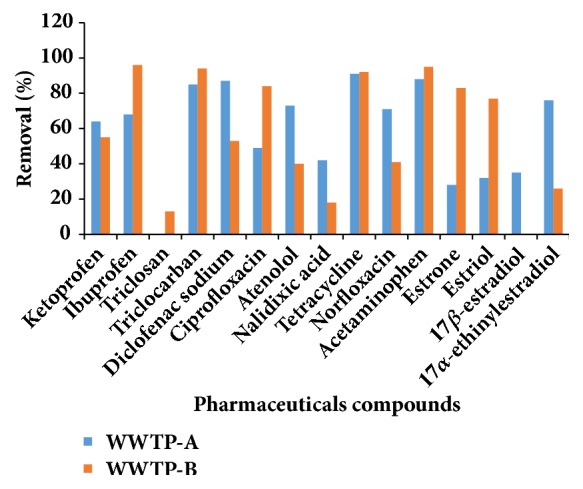
Average removal of pharmaceuticals in both WWTPs.

Taking into account the varying physicochemical properties of the selected pharmaceuticals and steroid hormones, it was expected that the WWTPs would exhibit different removal rates for these contaminants during the treatment processes. As shown in Figure 2, the PPCP removal rates ranged from 28 to 94% in WWTP-A, while the removal rates in WWTP-B ranged between 13 and 96%.

## 4. Discussion

### 4.1. Occurrence of PPCPs and Steroid Hormones in Influent Samples

Antibiotics are extensively used in both healthcare facilities. In fact, antibiotics are considered as one of the most commonly prescribed medications in the hospitals [20; 51]. However, the monitoring of the residuals of this category of therapeutic drugs is particularly necessary because of their contributions to the development of multidrug-resistant strains of microorganisms in municipal wastewaters as well as in the receiving water bodies [21]. Interestingly, three of the five PPCPs (CIP, NOR, and TCN) belonging to this group were detected in both WWTPs, while OFL and CAL were not detected.

In WWTP-A, the concentration of TCN varied from 1.09 to 45.38 *μ*g/L with an average concentration of 11.04 *μ*g/L, while a range of 3.80-75.81 *μ*g/L with an average concentration of 19.04 *μ*g/L was observed in WWTP-B. These results revealed that there is no significant difference in TCN concentration between the two WWTPs (p>0.05). Tetracycline (TCN) concentrations observed in the present study were higher than those previously reported in Hong Kong WWTPs by Li et al. [50], with a mean concentration of 0.270 *μ*g/L. Higher concentration of TCN was observed in hospital wastewater compared to municipal wastewater. Tetracycline (TCN) is normally used to treat urinary tract infections caused by certain bacteria [51].

The lowest mean concentrations were observed for norfloxacin (NOR), and were found to be 0.42 and 0.26 *μ*g/L for WWTP-A and WWTP-B, respectively. These results revealed that there is no significant difference in NOR concentration between the two WWTPs (p>0.05). The observed mean concentrations were also higher than those reported for this antibiotic (0.018 *μ*g/L) by Zorita et al. [52]. With respect to the concentrations of ciprofloxacin (CIP) in the WWTPs, a relatively lower mean concentration of 0.99 *μ*g/L was observed in WWTP-A compared to WWTP-B, whose mean concentration was 2.2 *μ*g/L. These results revealed that there is no significant difference in CIP concentration between the two WWTPs (p>0.05). These concentrations were similar to those previously reported for this antibiotic in some Canadian WWTPs [15] and were generally higher than those reported in municipal sewage treatment plants in Sweden [52].

Beta-blockers are another important class of therapeutic drugs that are frequently prescribed for the treatment of cardiovascular diseases and hypertension [53]. Atenolol (ATE) is a popular candidate of this group of pharmaceuticals. It was detected with a range of 1.08-8.34 *μ*g/L, having a total mean concentration of 4.41 *μ*g/L in WWTP-A, while its concentrations ranged from 0.41 to 2.54 *μ*g/L with a total mean concentration of 1.19 *μ*g/L in WWTP-B. These results revealed that ATE concentration at WWTP-A differed significantly from that detected in WWTP-B (p<0.05). The observed levels of atenolol in the present study were significantly lower than those reported for this compound in wastewater samples collected from the Northern Wastewater Treatment Works in KwaZulu-Natal Province, South Africa [43].

Triclosan (TCS) and triclocarban (TCC) are extensively used in a variety of consumer products because of their excellent antimicrobial and antifungal properties. These chemicals are commonly added to products such as soaps, disinfectants, toothpastes, body washes, and medical disinfectant, where they may contain between 0.1 and 2% of TCS or TCC by weight [46]. Triclosan (TCS) was found below the limit of detection in WWTP-A, while it was detected with a mean concentration of 0.11 *μ*g/L in WWTP-B. On the other hand, TCC was detected with total mean concentrations of 0.76 *μ*g/L and 0.61 *μ*g/L for WWTP-A and WWTP-B, respectively. These results revealed that there is no significant difference in TCC concentration between the two WWTPs (p>0.05). In comparison with the Canadian study, similar concentrations of TCC (0.56 *μ*g/L) were observed, whereas a relatively higher concentration of TCS (1.3 *μ*g/L) was observed than that in the present study [15].

Analgesics and anti-inflammatory drugs represent another group of pharmaceuticals that are highly utilised in isolation or may be combined with other formulations for the treatment of various disorders [54]. In the present study, all four target candidates (ACE, IBU, KET, and DIC) belonging to this therapeutic group were detected in both WWTPs. Among these pharmaceuticals, ACE had the highest concentrations corresponding to 49.79 and 24.07 *μ*g/L for WWTP-A and WWTP-B, respectively. This results revealed that there is no significant difference in ACE concentration between the two WWTPs (p>0.05). A similar concentration for ACE (59 *μ*g/L) in the influent of Northern Wastewater Treatment works in KwaZulu-Natal, South Africa was previously reported [43], but a lower concentration was reported in WWTP influent (2.953 *μ*g/L) in China [55]. Ibuprofen (IBU) was also detected in relatively high concentrations in the influent samples with mean concentrations of 16.44 and 14.39 *μ*g/L for WWTP-A and WWTP-B, respectively. In a related study, a similar concentration (19.7 *μ*g/L) was observed for IBU in one of the investigated influent samples [4]. These results revealed that there is no significant difference in IBU concentration between the two WWTPs (p>0.05). Similarly, the mean concentrations of ACE and IBU in influent samples in another study done (Lin et al. [56]) were 30.97*μ*g/L and 17.93 *μ*g/L, respectively, which were comparable to the findings in the present study. In contrast, a relatively lower concentration of IBU was detected in influent samples collected from the Darvill Wastewater Treatment Works which serves the Msunduzi Municipality and discharges its treated effluent into the Msunduzi River in KwaZulu-Natal [44]. Furthermore, the mean concentrations observed for IBU in the present study were also higher than those reported for similar matrices in Italy, Poland and Portugal [57–59].

Compared to other pharmaceuticals (ACE and IBU) in the analgesics and anti-inflammatory category, lower mean DIC concentrations of 2.34 *μ*g/L and 0.99 *μ*g/L were detected in WWTP-A and WWTP-B, respectively. Comparable mean concentrations of this compound had also been reported for influent samples collected from the city of Algiers [60]. Higher DIC concentrations were reported in WWTP influent (22.3 *μ*g/L) in KwaZulu-Natal [61].

Ketoprofen (KET) was found to have an average concentration of 0.39 *μ*g/L and 0.53 *μ*g/L in WWTP-A and WWTP-B, respectively. These results revealed that there is no significant difference in KET concentration between the two WWTPs (p>0.05). Similar results were reported in Italian WWTPs [57]. However, a low concentration of KET was observed among the group of WWTPs evaluated; the possible reason could be the low usage of KET in the particular hospital.

Oestrogens have both natural and synthetic origins [62]. The three major naturally occurring forms of oestrogens which are found in human urine are 17*β*-estradiol (E2) and its principal metabolites, estrone (E1) and estriol (E3). In addition, the synthetic oestrogenic compound, 17*α*-ethinylestradiol (EE2), is frequently used as the main ingredient in many oral contraceptives [63]. In WWTP-A, the concentrations of E1 varied from 0.013 to 0.053 *μ*g/L with a mean concentration of 0.031 *μ*g/L, while a range of 0.004-0.04 *μ*g/L with a mean concentration of 0.018 *μ*g/L was observed in WWTP-B. These results revealed that there is no significant difference in E1 concentration between the two WWTPs (p>0.05).

A similar concentration of E1 (0.023 *μ*g/L) was detected in hospital wastewater influent in Oslo, Norway [28]. However, the observed levels in the present study were significantly higher than those reported for E1 in the hospital wastewater in Korea [38]. Similarly, the concentration of E2 in WWTP-A varied from 0.008 to 0.035 *μ*g/L with a mean concentration of 0.022 *μ*g/L whereas a range of 0.001-0.047 *μ*g/L with a mean concentration of 0.018 *μ*g/L was observed in WWTP-B. A similar concentration of E2 (0.02 *μ*g/L) has been reported in hospital wastewater influent in Oslo, Norway [28], although the observed levels were generally lower than the E2 concentrations in the influent of WWTPs in China receiving mainly domestic wastewater [64].

Estriol (E3) was the second most abundant hormone detected in the analysed influent samples. It was detected in a concentration range of 0.134-1.480 *μ*g/L with a total mean concentration of 0.463 *μ*g/L in WWTP-A, while its total mean concentration in WWTP-B was 0.257 *μ*g/L with a concentration range of 0.027-0.512 *μ*g/L. These results revealed that there is no significant difference in E3 concentration between the two WWTPs (p>0.05). The detection of elevated levels of this hormone in wastewater is presumably associated with its high excretion rates by humans [64]. The concentrations detected in the present study are similar to the mean concentration reported for E3 (0.328 *μ*g/L) in domestic WWTP influent in Tunisia [65].

In WWTP-A, the concentration of EE2 varied from 2.654 to 9.833 *μ*g/L with an average concentration of 5.601 *μ*g/L while a range of 0.881-1.041 *μ*g/L with an average concentration of 0.923 *μ*g/L was observed in WWTP-B. These results revealed that the EE2 concentration in WWTP-A differed significantly from that observed in WWTP-B (p<0.05). In contrast, EE2 was not detected in hospital wastewater in Korea [38]. High concentrations of EE2 in influent streams entering WWTPs may be due to the fact that EE2 is a major ingredient in contraceptive pills.

### 4.2. Occurrence of PPCPs and Steroid Hormones in Effluent Samples

The concentrations of the seventeen (17) target PPCPs in the effluent samples of both WWTPs are presented in Table 6 and Table 7. Similar to the observed trend in the influent samples, 14 of the 17 target PPCPs were detected in the effluent samples collected from the two WWTPs that were evaluated in this study. As observed in WWTP-B influent samples, OFL, CAL, and BF were also not detected in WWTP-B effluent samples, whereas TCS and E2, in addition to those target PPCPs that were not detected in WWTP-B, were not detected in any effluent sample collected from WWTP-A. However, all target PPCPs (where detected) were observed at lower concentrations in the effluent than in the influent samples in both of the WWTPs that were evaluated, thus indicating the significant removal efficiency of these wastewater treatment processes.

Among the antibiotics, only OFL and CAL were not detected in effluent samples from both WWTPs. Triclosan (TCN) was detected at low concentrations with a mean value of 0.95 *μ*g/L in WWTP-A, whereas a relatively higher mean concentration (1.49 *μ*g/L) was observed in WWTP-B. These results revealed that there is no significant difference in TCN concentration between these two WWTPs (p>0.05).

Both CIP and NOR were detected at very low concentrations in these two WWTPs. In WWTP-A, the overall mean concentrations of 0.51 *μ*g/L and 0.12 *μ*g/L were observed for CIP and NOR, respectively. In WWTP-B, similar concentrations were observed for CIP (0.35 *μ*g/L) and NOR (0.15 *μ*g/L). The observed concentrations in the present study were comparable to those reported for NOR in the effluent of some WWTPs in China [66], and for CIP in effluent samples in the USA [67]. These results revealed that there is no significant difference in CIP and NOR concentration between these two WWTPs (p>0.05).

The beta blocker ATE was found at a higher concentration in WWTP-A (1.19 *μ*g/L) compared to that detected in WWTP-B (0.71 *μ*g/L). These results revealed that there is no significant difference in ATE concentration between these two WWTPs (p>0.05). Compared to the levels observed in the present study, lower concentrations of ATE have been reported in hospital WWTP effluents (0.055 *μ*g/L) in Saudi Arabia [68]. The high concentrations of ATE in WWTP-A effluent may be related to its high usage by the hospital.

Triclosan (TCS) was not detected in WWTP-A while it was found at very low concentrations in WWTP-B (0.09 *μ*g/L). Compared to the levels observed in the present study, higher concentrations of TCS have been reported in municipal WWTP effluents (0.108 *μ*g/L) in Canada [69]. Triclocarban (TCC) was also detected at very low concentrations in both WWTPs, with mean concentrations of 0.11 and 0.04 *μ*g/L for WWTP-A and WWTP-B, respectively. These results revealed that there is no significant difference in TCC concentration between these two WWTPs (p>0.05).

Among the analgesics, ACE was still detected at fairly high concentrations with mean concentrations of 6.1 and 1.24 *μ*g/L for WWTP-A and WWTP-B, respectively. These results revealed that there is no significant difference in ACE concentration between these two WWTPs (p>0.05).

Ibuprofen (IBU) was also detected at high concentrations with a mean concentration of 5.25 *μ*g/L for WWTP-A, although a much lower mean concentration (0.51 *μ*g/L) was observed for WWTP-B. These results revealed that there is no significant difference in IBU concentration between the two WWTPs (p>0.05). The observed concentrations of IBU were generally lower than those detected in the Darvill Wastewater Treatment Works effluent being discharged into the Msunduzi River in KwaZulu-Natal [44]. With respect to the levels of DIC, a similar concentration range was observed in both investigated WWTPs. These results revealed that there is no significant difference in DIC concentration between these two WWTPs (p>0.05).

These concentrations were similar to those previously reported in WWTP effluents in France (Marseilles) [70]. However, the observed concentrations in the present study were much lower than the levels reported in WWTP effluent in KwaZulu-Natal [71]. In both of the WWTPs that were investigated in this study, KET was detected at lower concentrations with mean values of 0.14 and 0.23 *μ*g/L for WWTP-A and WWTP-B, respectively. These results revealed that there is no significant difference in KET concentration between these two WWTPs (p>0.05).

All the target oestrogens were detected in effluent samples collected from WWTP-A, while only E2 was not detected in WWTP-B effluent samples. The levels of E1 observed in WWTP-A ranged from 0.007 to 0.041 *μ*g/L and these were relatively higher than those detected in effluent samples collected from WWTP-B. The results revealed that the E1 concentration in WWTP-A differed significantly from that observed in WWTP-B (p<0.05). A similar E1 concentration (0.0041 *μ*g/L) was reported by Pauwels & coworkers [71] in effluent samples from hospital WWTPs in Belgium.

The naturally occurring oestrogen, 17*β*-estradiol (E2), was not detected in WWTP-B effluent while it was found in WWTP-A effluent in a range of 0.008-0.019 *μ*g/L with a mean concentration of 0.014 *μ*g/L. Similarly, E3 was detected in WWTP-A in a range of 0.111-0.539 *μ*g/L and a total mean concentration of 0.233 *μ*g/L. In WWTP-B, its total mean concentration was 0.043 *μ*g/L with a concentration range of 0.01-0.065 *μ*g/L. The results revealed that the E2 concentration in WWTP-A effluent differed significantly from that detected in WWTP-B effluent (p<0.05).

The synthetic oestrogen, 17*α*-ethinylestradiol (EE2), was still detected in relatively high concentrations in both WWTPs. In WWTP-A effluent, the concentration of EE2 varied from 0.448 to 4.608 *μ*g/L with a mean concentration of 1.344 *μ*g/L, while a range of 0.524-0.884 *μ*g/L with a mean concentration of 0.681 *μ*g/L was observed in WWTP-B effluent. These results revealed that there is no significant difference in EE2 concentration between the two WWTPs (p>0.05). However, a lower EE2 concentration (0.0162 *μ*g/L) in domestic WWTP effluent samples in China has been reported [63].

### 4.3. Removal Efficiencies of Pharmaceuticals and Steroids Hormones

Among the antibiotics, the highest removal rate was observed for TCN, which was approximately 91% for both WWTPs. Higher CIP removal was observed in WWTP-B (84%) than in WWTP-A (49%). Similarly, the NOR removal rate was relatively higher in WWTP-A (71%) than in WWTP-B (41%). In general, our findings can be compared with those previously reported for the removal of antibiotics in different WWTPs in Taiwan where their removal rates were found to be between 0 and 82% [72]. Sorption onto sludge is usually considered as the main elimination mechanism for quinolone antibiotics [73, 74]. Regardless of their negative octanol/water partition coefficient (*K*_ow_), NOR and CIP have a high affinity for sorption as a result of their zwitterionic character (pK_aCOOH_ = 5.9-6.4; pK_aNH2_ = 7.7-10.2) [74].

The average removal rate for ATE was found to be higher in WWTP-A (73%) than in WWTP-B (40%). A somewhat similar finding was reported for ATE where its average removal rate was found to be 60% in selected WWTPs in Finland [75]. Because ATE has a low octanol/water partition coefficient (log *K*_ow_ = 0.16), its removal from aqueous waste streams is not attributable to adsorption to sludge but to biodegradation [76].

Triclosan (TCS) was not detected in WWTP-A while a total removal rate of 13% was observed in WWTP-B. The estimated removal rate in this study was within the range previously reported for TCS in a related study [77]. However, this was somewhat lower than the removal rates reported for TCS elsewhere which were generally higher than 60% [49, 78, 79]. Despite its relatively high log *K*_ow_ value (4.76), it was still poorly removed by WWTP-B. The probable explanation for its low removal rate is that it could be due to its persistence on the sludge particles, as well as the absence of TCS degrading bacteria in the microbial community of the activated sludge [77]. Triclosan (TCC) removal rates of 85% and 94% were observed for WWTP-A and WWTP-B, respectively. High TCC removal rates can be attributed to its high octanol/water partition coefficient (log *K*_ow_ = 4.9) and the presence TCC degrading bacteria in the sludge particles, therefore sorption to sludge was demonstrated as its major elimination mechanism in activated sludge treatment processes [80].

Acetaminophen (ACE) was present at high concentrations in the influent samples and more than 80% of these were removed in both WWTPs. The high ACE removal rate observed was in agreement with similar findings reported elsewhere [75, 81–83]. Considering its low log *K*_ow_ value (0.46), ACE is not expected to be adsorbed onto sludge particles; hence microbial degradation is considered to be an important route for its elimination [84]. Good removal rates were observed for IBU even though high concentrations were detected in the influent samples. Its average removal rate was found to be 68% for WWTP-A and nearly 100% for WWTP-B. These results are in agreement with those reported elsewhere [11, 37, 60, 85]. A higher removal rate was observed for DIC in WWTP-A (87%), while a relatively low removal rate was obtained in WWTP-B (53%). In contrast, a much lower removal rate for IBU (<21%) was reported at WWTPs in Greece [37]. The KET removal rate was found to be 64% and 55% in WWTP-A and WWTP-B, respectively. Lindqvist et al. (2005) [4] investigated the treatment efficiency of seven sewage treatment plants in Finland and found an average KET removal rate of 78%, which was marginally higher than the KET removal efficiency observed in this study. Good removal rates for KET, IBU and DIC can be attributed to their relatively high octanol/water partition coefficient (log *K*_ow_ = 3-4), which often favours sorption to solids as the primary elimination mechanism for these contaminants.

The mean removal rate for E1 in WWTP-B was 83%. On the other hand, WWTP-A employing the same treatment process was observed to have an average removal rate of 28%, thus suggesting that the operating conditions and reactor configuration may have influenced the treatment performance. The naturally occurring oestrogen, 17*β*-estradiol (E2), was not detected in WWTP-B effluent while a mean removal efficiency of 35% was observed in WWTP-A. However, this value is lower than the E2 removal efficiency reported in a Brazilian WWTP which was higher than 90% [86].

The mean removal rate for EE2 in WWTP-A was 76%. This result is in agreement with Pessoa et al. [86], who reported a similar removal rate (75.6%) for EE2. Contrary to the above-mentioned results for WWTP-A, WWTP-B yielded a low removal of 26%. The difference in terms of the EE2 removal rates between WWTP-A and WWTP-B might be attributed to the possible difference in the influx of EDCs into the respective WWTPs. The same pattern was observed for E3 where a higher removal rate (77%) was observed in WWTP-B, whereas its removal rate in WWTP-A was 32%. Generally, steroid hormones have high log *K*_ow_ values and therefore sorption to solids is expected to be the most dominant elimination mechanism for their removal during wastewater treatment processes.

### 4.4. Comparison of WWTPs Evaluated

Our findings revealed that, over and above the fact that there is a wide variability in terms of the concentrations of the PPCPs and the steroid hormones, there is also a clear distinction with respect to removal efficiencies in both WWTPs. For instance, some compounds such as acetaminophen and tetracycline are removed during the treatment process to the same degree in both WWTPs, whereas the removal of others such as ciprofloxacin and norfloxacin differed considerably in both WWTPs. In this study, the two hospital wastewater treatment plants basically applied the same treatment processes, which consisted of an activated sludge process with a carrousel-type biological reactor, followed by secondary settling tanks and maturation ponds as well as disinfection units. In general, higher concentrations of the target PPCPs and the steroid hormones were detected in the influent samples of WWTP-A compared to WWTP-B. The only exceptions were observed for acetaminophen, ibuprofen, and tetracycline where relatively similar concentrations were detected in both plants. The behaviour of the target compounds in these WWTPs may have been influenced by a number of factors such as (i) the type and size of the investigated health facilities, the average daily influx of wastewater, water consumption rates, and availability of sanitation facilities; (ii) possible difference in pharmaceutical consumption patterns between the two healthcare facilities discharging wastewater into the two WWTPs that were evaluated; and (iii) possible distinctive removal efficiencies which may be dependent on several design and operating factors such as reactor configuration, variations in feed concentration and flow rate in the biological tank, temperature, sludge retention time (SRT), and hydraulic retention time (HRT) as discussed in Verlicchi et al. [30], as well as the availability of skilled personnel required to effectively manage the treatment processes for optimal performance.

## 5. Conclusions

Fourteen of the seventeen target PPCPs in this study were detected in influent and effluent samples collected from both WWTPs. Acetaminophen, ibuprofen, and tetracycline were present at high concentrations (10-50 *μ*g/L) in the influent samples. The carrousel-type activated sludge system decreased the influent concentrations of all target PPCPs by 40–98% before their eventual discharge, except for triclosan which was shown to have a low removal rate (13%). Despite the high removal efficiencies observed for acetaminophen, ibuprofen, and tetracycline, they were still present in the effluent samples at high concentrations, usually exceeding 1 *μ*g/L, which may present a serious threat to the growth and survival of aquatic organisms in the receiving water bodies. It is therefore imperative to subject the treated effluent to further treatment processes to ensure that the effluent can be safely discharged into the environment without causing harm. The adoption of advanced treatment processes such as the use of activated carbon should be considered to minimise the contamination in the receiving water bodies in order to ensure that the water may be safely reused in the near future.

## Figures and Tables

**Table 1:** Concentrations of different types of pharmaceuticals in HWW from the reviewed studies.

**Compound**	**HWW (** ***μ*** **g/L)**	
	**Range**	**Reference**
**Antibiotics**		

Ciprofloxacin	0.85-2	Brown et al., 2006 [21]
	2.0-83	Kümmerer et al., 2001 [22]
	3.6-101	Lindberg et al., 2004 [23]

Ofloxacin	2.905	Chang et al., 2010 [24]
	6.67	Gros et al., 2013 [25]
	2.20	Passerat et al*., *2010 [26]

Nalidixic acid	0.186	Lin et al., 2008 [27]

	<0.002-<0.005	Gros et al.,2013 [25]
Tetracycline	<0.015-4.178	Thomas et al*.,* 2007 [28]
	<0.002-0.455	Lin & Tsai, 2009 [29]
	<0.007-0.033	Verlicchi et al., 2012a [30]

Chloramphenicol	<0.004-0.036	Verlicchi et al., 2012a [30]
	0.001	Lin et al*.,* 2008 [31]
	<0.5	Ohlsen et al., 2003 [32]

**Hormones**		
Estrone	0.007-0.04	Thomas et al*., *2007 [28]
	<0.01	Lin et al., 2008 [31]
	0.025-0.415	Lin & Tsai, 2009 [29]

17*β*-estradiol	<0.003-0.072	Thomas et al.,2007 [28]
	<0.025-0.23	Thomas et al., 2007 [28]
	<0.01	Lin et al., 2008 [31]

Estriol	0.18-0.785	Thomas et al.,2007 [28]
	4.651	Lin et al., 2008 [31]

17*α*-ethinylestradiol	<0.003	Thomas et al., 2007 [28]
	<0.025-0.432	Lin & Tsai, 2009 [29]
	<0.0004	Perrodin et al., 2013 [33]

**Beta-blockers**		
Atenolol	0.045-0.0053	Langford & Thomas, 2009 [34]
	0.1-122	Gómez et al., 2006 [35]
	2.2-6.6	Verlicchi et al*.,* 2012b [36]

**Disinfectants**		
Triclosan	<0.044	Kosma et al., 2010 [37]

**Lipid modifying agents**		
Bezafibrate	<0.001-2.9	Verlicchi et al., 2012 [30]

**Analgesics**		
Diclofenac	0.06-1.9	Gómez et al., 2006 [35]
	0.17-0.53	Verlicchi et al., 2012 [30]
	0.028-6.88	Sim et al., 2011 [38]

Acetaminophen	0.5-29	Gómez et al., 2006 [35]
	1.4-5.9	Verlicchi et al., 2012b [36]
	0.271-63.1	Sim et al., 2011 [37]

Ibuprofen	7-8.93	Kosma et al., 2010 [37]
	1.5-151	Gómez et al., 2006 [35]
	0.069-8.957	Thomas et al., 2007 [28]

Ketoprofen	0.2-0.35	Langford *& *Thomas, 2009 [34]
	1.1-9.8	Verlicchi et al., 2012 [30]
	<0.01-0.23	Lin & Tsai, 2009 [29]

**Table 2:** Selected pharmaceuticals and their physicochemical properties.

Pharmaceutical class	Compound	MW g/mol	Log K_OW_
Antibiotics	Ciprofloxacin	331.35	0.28
	Ofloxacin	361.4	-0.39
	Norfloxacin	319.34	-0.13
	Tetracycline	444.44	-1.3
Beta-blockers	Atenolol	266.3	0.16
Disinfectants	Triclosan	289.54	4.76
	Triclocarban	315.58	4.9
Analgesics/anti-inflammatory drugs	Diclofenac	294	4.51
	Acetaminophen	151.2	0.46
	Ibuprofen	206.3	3.97
	Ketoprofen	254.3	3.12
Lipid modifying agents	Bezafibrate	361.8	4.25
Steroid hormones	Estrone	270.4	3.13
	Estriol	288.4	2.45
	17*β* estradiol	272.4	4.01
	17*α*- ethinyl estradiol	296.4	4.2

Physical and chemical information was obtained from Ratola et al., 2012 [45]; Shaver, 2011 [46].

**Table 3:** Mass spectrometry parameters for pharmaceutical and hormone analysis (quantitative ion marked in **bold**; confirmation ion).

**Compound**	**Precursor *m/z***	**Product *m/z***	**Cone voltage (V)**	**Collision energy (eV)**
**Negative mode**				
Ketoprofen (KET)	253.3	**209.29**	15	8
Ibuprofen (IBU)	205.4	**161.3**	17	9
Bezafibrate (BEZ)	360.2	**274**; 153.95	13	16
Triclosan (TCS)	286.8	**35**; 141.8	22	11
Triclocarban (TCC)	313.1	**159.9**; 126.05	12	13
Chloramphenicol	321.1	**152**; 257.05	16	19
Estrone (E1)	269.2	**145.10**; 143.05	14	46
17*β*-estradiol (*β*-E2)	271.2	**145.10; **183.10	14	47
Estriol (E3)	287.2	**143.05; **171.15	15	43
17*α*-ethinylestradiol (EE2)	295.3	**145.05;** 159.05	11	47
**Positive mode**				
Tetracycline (TCN)	444.9	**410**; 154.1	-13	-21
Nalidixic acid (NAL)	232.9	**215.05; **187	-18	-16
Atenolol (ATE)	267	**145; **190	-14	-29
Acetaminophen (ACE)	151.9	**110.1**; 65.15	-11	-24
Ofloxacin (OFL)	362	**318.1**; 261.05	-14	-22
Norfloxacin (NOR)	320	**302.05**; 231.05	-16	-22
Ciprofloxacin (CIP)	332	**314.1**; 231	-13	-23
Diclofenac sodium salt (DIC)	318	**23.2**; 58.75	-12	-15

**Table 4:** Method validation parameters.

**Compound**	***R*** ^***2***^	%**RSD**	**LOD (** ***μ*** **g/L)**	**LOQ (** ***μ*** **g/L)**
^**13**^ **C3-Caffeine**	0.9995	1	0.03	0.102
**CIP**	0.9997	26.28	0.06	0.18
**OFL**	0.9997	18.07	0.05	0.15
**NOR**	0.9991	8.49	0.04	0.12
**TCN**	0.9994	24.72	1.66	5.02
**ATE**	0.9979	7.34	0.4	1.22
**TCS**	0.9999	101.92	0.17	0.51
**TCC**	0.9997	101.92	0.07	0.22
**DIC**	0.9992	32.08	0.05	1.23
**ACE**	0.9999	28.6	0.13	0.38
**IBU**	0.9999	12.51	0.19	0.59
**KET**	0.9991	67.5	0.07	0.23
**E1**	0.9996	25.97	0.79	2.41
**E3**	0.9988	10.22	1.3	3.93
**β** **-E2**	0.9993	81.07	0.38	1.71
**EE2**	0.9992	23.66	0.6	1.82
^**13**^ **C3-E1**	0.9995	4.19	0.11	0.33

LOD: limit of detection; LOQ: limit of quantification; RSD: relative standard deviation.

**Table 5:** Percentage recovery of pharmaceuticals.

**Compounds**	**Concentration level**	
	**10 ng/mL **	**100 ng/L **
E1	146	101
E3	88	92
E2	70	113
EE2	75	90

**Table 6:** Overall concentrations of pharmaceuticals in influents and effluents (*μ*g/L) in WWTP-A.

WWTP-A	Influent		Effluent	
	Mean conc.	Min-Max conc.	Mean conc.	Min-Max conc.
**Antibiotics**				
CIP	0.99 ± 0.80	0.12-2.00	0.51 ± 0.57	0.08-1.40
OFL	ND	ND	ND	ND
NOR	0.42 ± 0.62	0.10-1.53	0.12 ± 0.13	0.03-0.35
CAL	ND	ND	ND	ND
TCN	11.04 ± 19.22	1.09-45.38	0.95 ± 0.38	0.52-1.43
**Beta-blockers**				
ATE	4.41 ± 2.76	1.08-8.34	1.19 ± 1.27	0.33-3.22
**Disinfectants**				
TCS	ND	ND	ND	ND
TCC	0.76 ± 0.67	0.23-1.75	0.11 ± 0.19	0.01-0.46
**Analgesics/anti-inflammatory drugs **				
DIC	2.38 ± 4.46	0.12-10.34	0.3 ± 0.27	0.07-0.75
ACE	49.79 ± 40.43	21.27-119.50	6.1 ± 7.50	<LOQ-11.39
IBU	16.44 ± 23.23	0.33-53.40	5.25 ± 7.35	<LOQ-13.66
KET	0.39 ± 0.38	<LOQ-0.65	0.14 ± 0.08	<LOQ-0.24
**Lipid modifying agents**				
BF	ND	ND	ND	ND
**Hormones**				
E1	0.031 ± 0.02	0.013-0.053	0.023 ± 0.014	0.007-0.041
E3	0.463 ± 0.57	0.134-1.480	0.233 ± 0.157	0.111-0.539
*β*-E2	0.022 ± 0.02	0.008-0.035	0.014 ± 0.004	0.008-0.0191
EE2	5.601 ± 2.66	2.654-9.833	1.344 ± 1.827	0.448-4.608

ND: not detected.

**Table 7:** Overall concentrations of pharmaceuticals in influents and effluents (*μ*g/L) in WWTP-B.

WWTP-B	Influent		Effluent	
	Mean conc.	Min-Max conc.	Mean conc.	Min-Max conc.
**Antibiotics**				
CIP	2.2 ± 3.89	0.12-9.11	0.35 ± 0.52	0.06-1.28
OFL	ND	ND	ND	ND
NOR	0.26 ± 0.31	0.07-0.82	0.15 ± 0.19	0.02-0.44
CAL	ND	ND	ND	ND
TCN	19.11 ± 31.73	3.80-75.81	1.49 ± 1.12	0.48-3.22
**Beta-blockers**				
ATE	1.19 ± 0.89	0.41-2.54	0.71 ± 0.82	0.15-2.15
**Disinfectants**				
TCS	0.11 ± 0.19	0.00-0.40	0.09 ± 0.12	0.00-0.27
TCC	0.61 ± 0.38	0.06-0.97	0.04 ± 0.03	0.00-0.06
**Analgesics/anti-inflammatory drugs **				
DIC	0.99 ± 1.59	0.15-3.82	0.47 ± 0.63	0.10-1.58
ACE	24.07 ± 22.47	11.06-57.65	1.24 ± 1.19	<LOQ-2.09
IBU	14.39 ± 27.40	1.09-63.37	0.51 ± 0.61	0.02-1.46
KET	0.53 ±0.25	<LOQ-0.70	0.23 ± 0.32	<LOQ-0.61
**Lipid modifying agents**				
BF	ND	ND	ND	ND
**Hormones**				
E1	0.018 ± 0.014	0.004-0.040	0.004 ± 0.002	0-6
E3	0.257 ± 0.199	0.027-0.512	0.043 ± 0.024	0.01-0.065
Β-E2	0.0178 ± 0.020	0.001-0.047	<LOQ	
EE2	0.923 ± 0.067	0.881-1.041	0.681 ± 0.130	0.524-0.884

ND: not detected.

## Data Availability

The data used to support the findings of this study are included within the article.
